# Renal chymase-dependent pathway for angiotensin II formation mediated acute kidney injury in a mouse model of aristolochic acid I-induced acute nephropathy

**DOI:** 10.1371/journal.pone.0210656

**Published:** 2019-01-11

**Authors:** Wen-Yeh Hsieh, Teng-Hsiang Chang, Hui-Fang Chang, Wan-Hsuan Chuang, Li-Che Lu, Chung-Wei Yang, Chih-Sheng Lin, Chia-Chu Chang

**Affiliations:** 1 Division of Pulmonary Medicine, Department of Internal Medicine, Hsinchu Mackay Memorial Hospital, Hsinchu, Taiwan; 2 Division of Nephrology, Department of Internal Medicine, Changhua Christian Hospital, Changhua, Taiwan; 3 Department of Biological Science and Technology, National Chiao Tung University, Hsinchu, Taiwan; 4 Division of Endocrinology, Department of Internal Medicine, Hsinchu Mackay Memorial Hospital, Hsinchu, Taiwan; 5 Division of Nephrology, Department of Internal Medicine, Shin Kong Wu Ho-Su Memorial Hospital, Taipei, Taiwan; 6 Division of Nephrology, Department of Internal Medicine, National Taiwan University Hospital Hsinchu Branch, Hsinchu, Taiwan; 7 Center for Intelligent Drug Systems and Smart Bio-devices (IDS2B), National Chiao Tung University, Hsinchu, Taiwan; 8 School of Medicine, Chung-Shan Medical University, Taichung, Taiwan; 9 Department of Environmental and Precision Medicine Laboratory, Changhua Christian Hospital, Changhua, Taiwan; 10 Department of Nutrition, Hungkuang University, Taichung, Taiwan; 11 Department of Internal Medicine, Kuang Tien General Hospital, Taichung, Taiwan; Max Delbruck Centrum fur Molekulare Medizin Berlin Buch, GERMANY

## Abstract

Angiotensin-converting enzyme (ACE) is the primary enzyme that converts angiotensin I (Ang I) to angiotensin II (Ang II) in the renin-angiotensin system (RAS). However, chymase hydrates Ang I to Ang II independently of ACE in some kidney diseases, and it may play an important role. The present study investigated whether chymase played a crucial role in aristolochic acid I (AAI)-induced nephropathy. C57BL/6 mice were treated with AAI via intraperitoneal injection for an accumulated AAI dosage of 45 mg/kg body weight (BW) (15 mg/kg BW per day for 3 days). The animals were sacrificed after acute kidney injury development, and blood, urine and kidneys were harvested for biochemical and molecular assays. Mice exhibited increased serum creatinine, BUN and urinary protein after the AAI challenge. Significant infiltrating inflammatory cells and tubular atrophy were observed in the kidneys, and high immunocytokine levels were detected. Renal RAS-related enzyme activities were measured, and a significantly increased chymase activity and slightly decreased ACE activity were observed in the AAI-treated mice. The renal Ang II level reflected the altered profile of RAS enzymes and was significantly increased in AAI-treated mice. Treatment of AAI-induced nephropathic mice with an ACE inhibitor (ACEI) or chymase inhibitor (CI; chymostatin) reduced renal Ang II levels. The combination of ACEI and CI (ACEI+CI) treatment significantly reversed the AAI-induced changes of Ang II levels and kidney inflammation and injuries. AAI treatment significantly increased renal p-MEK without increasing p-STAT3 and p-Smad3 levels, and p-MEK/p-ERK1/2 signalling pathway was significantly activated. CI and ACEI+CI treatments reduced this AAI-activated signaling pathway. AAI-induced nephropathy progression was significantly mitigated with CI and ACEI+CI treatment. This study elucidates the role of RAS in the pathogenesis of AAI-induced nephropathy.

## Introduction

Aristolochic acid nephropathy (AAN) is a rapidly progressive interstitial nephritis that leads to urothelial malignancy, end-stage renal disease and irreversible kidney failure. AAN was originally reported in Belgium in a group of patients who ingested slimming pills that contained powdered root extracts of Chinese herbs [1,2]. The incidence of AAN is likely high because of the presence of aristolochic acid (AA) in herbal remedies and lack of awareness of the disease [3]. AA is derived from the *Aristolochia* species, and it is the active principle agent in slimming pills. AA is a mixture of structurally related nitro-phenanthrene carboxylic acids, which are primarily composed of aristolochic acid I (AAI) and aristolochic acid II (AAII) [4]. Among these, AAI has been proven as the major factor of the nephrotoxicity associated with AAI-induced nephropathy [3,5,6]. Renal histology in the chronic pathology of AAN reveals the formation of tubulointerstitial fibrosis (TIF) and tubular atrophy [7]. Renal microvasculature injury and an imbalance of endothelial vasoactive agents may lead to fibrosis in AAN [3]. Sclerosis of glomeruli are also observed [8]. Animal models of AAN are widely used in investigations of renal toxicity of Aristolochia and Asarum genus herbs [9,10], and exhibit similar pathological characteristics as human chronic kidney diseases. Animal models of AAN have been used for the two past decades to examine the underlying molecular and cellular mechanisms involved in AAN pathogenesis [3]. AAI-induced rodent models of acute or chronic kidney injury/disease models are well-established [9]; however, information on the disease mechanisms of AAI-induced acute kidney injuries related to the dysregulation or imbalance of the renin-angiotensin system (RAS) are not known.

Angiotensin-converting enzyme (ACE) is the primary and classical enzyme that converts angiotensin I (Ang I) to angiotensin II (Ang II) in the renin-angiotensin system of cardiovascular and renal systems [11]. Unbalanced RAS and an abnormally activated ACE/Ang II axis are the primary effectors that contribute to the onset and progression of renal damage [12]. Abnormally excessive local Ang II may also directly contribute to the acceleration of renal damage via sustaining cell growth, inflammation, and fibrosis [13]. Clinical therapy for renal diseases generally includes angiotensin-converting enzyme inhibitor (ACEI) and Ang II receptor blockers (ARBs) to decrease ACE/Ang II activation and ameliorate disease development. However, the results of these treatments vary depending on the person and disease [14,15]. The treatment outcome of the combination of ACEIs and ARBs is controversial [16,17]. Therefore, the need for new therapeutic targets is fueled by the failure of traditional RAS blockade, such as the direct renin inhibitor aliskiren and chymase inhibitors.

Chymase is a serine protease that primarily converts Ang I to Ang II via an ACE-independent pathway [18]. The evidence has suggested that chymase is an alternative pathway of ACE conversion and Ang II formation in tissues [19], and it exhibits a catalytic efficiency 20-fold greater than ACE [20]. Chymase is weakly expressed in glomeruli and vascular smooth muscle cells in normal human kidney, and it is markedly upregulated in diabetic kidneys [21]. Renal chymase ameliorated renal TIF in unilateral ureteral obstruction [22]. In diabetic nephropathy rodent models, chymase inhibition protected diabetic rats from renal lesions [23], and chymase released TGF-β1 from the extracellular matrix (ECM) via specific proteolytic cleavage of the latent TGF-β binding protein to increase cellular inflammation [24]. Many interventions that inhibit RAS activity are renoprotective and may retard the progression of chronic nephropathies, but several studies suggested that chymase played an important role in some renal diseases [21–24]. Chymase plays a pivotal role in the pathogenesis of renal disease and kidney injury. Therefore, the present study examined the effects of the inhibition of chymase-induced Ang II formation in acute kidney injury in mice with AAI-induced nephropathy.

## Methods and materials

An animal model of acute nephropathy was performed in C57BL/6 mice. Mice were treated with AAI via intraperitoneal (i.p.) injection, and the accumulated AAI dosages was 45 mg/kg BW (body weight) (15 mg/kg BW per day for 3 days). Animals were sacrificed 24 hours after acute nephropathy development, and blood and kidneys were harvested for further biochemical and molecular assays. Blood and tissue samples were immediately isolated after anesthetization to reduce the physiological effects of the anesthesia, including RAS elements.

Mice were randomly divided into five groups (n = 9 for each group) and received the following treatments: (1) Control group—mice treated with saline; (2) AAI group—mice treated with i.p. AAI (15 mg/kg BW per day for 3 days); (3) AAI/ACEI group—mice treated with i.p. AAI + i.p. Captopril (10 mg/kg BW per day for 3 days); (4) AAI/CI (chymase inhibitor) group—mice treated with i.p. AAI + i.p. Chymostatin (10 mg/kg BW per day for 3 days); and (5) AAI/ACEI+CI group—mice treated with i.p. AAI + i.p. Captopril (10 mg/kg BW per day for 3 days) + i.p. Chymostatin (10 mg/kg BW per day for 3 days). AAI (#A5512), Captopril (ACEI; #C4042) and Chymostatin (#C7268) were purchased from Sigma-Aldrich (St. Louis, MO, USA).

All mice were maintained in a specific pathogen-free facility with free access to food and water. Animal care and all experimental procedures were performed in accordance with the Guideline for Animal Use Protocol National Chiao Tung University with approval of the Institutional Animal Care and Use Committee (permission number: NCTU-IACUC-103013). Mice were routinely checked twice daily for signs of illness. Mice that were likely to reach the humane endpoint prior to the next monitoring event (i.e., 20% body weight loss, hair removal or behavioral disorder) were immediately euthanized using CO_2_. None of the mice were found dead. Forty-five mice were humanely euthanized via exsanguination under anesthesia for tissue collection, and every effort was made to minimize distress and suffering. All animal care workers had over 2 years of experience working with laboratory animals.

### Sample preparation and Western blot

Kidney samples for use in biochemical and molecular analyses were prepared as described previously [25]. Organ samples were collected after animal sacrifice and homogenized 3 times in the lysis buffer PRO-PREPTM Protein Extraction Solution (iNtRON Biotechnology, Kyungki-Do, Korea). Samples were centrifuged at 13,000 x *g* for 10 min for separation of supernatants and pellets. The total amount of protein in the homogeneous extract was measured using the Bradford dye binding assay (Bio-Rad Laboratories, Hercules, CA, USA). The supernatants were aliquoted and stored at -80°C until further use.

Kidney homogenates with an equivalent protein content of 25 μg protein were electrophoresed on 12% SDS-PAGE gels and transferred to polyvinylidene fluoride membranes (Immobilon-PTM; Millipore, Bedford, MA, USA). Primary antibodies against phosphor-STAT3 (p-STAT3), p-ERK1/2, p-JNK, p-p38, p-Smad, p-MEK and β-actin were obtained from Genetex (Irvine, CA, USA) or Cell Signaling Technology (Beverly, MA, USA). Chemiluminescence substrates were visualized using enhanced chemiluminescence detection and a luminescence image system (LAS-3000; Fuji Film, Stamford, CT, USA). Bands on the images were detected at the anticipated location based on size. Band intensity was quantified using Scion Image software (Scion, Frederick, MD, USA). The levels of p-STAT3, p-ERK1/2, p-JNK, p-p38, p-Smad, p-MEK, p-ERK1/2 and p-STAT3 were normalized to β-actin.

### Measurements of ACE and ACE2 activity

Renal ACE and angiotensin-converting enzyme 2 (ACE2) activities were assayed using the fluorogenic substrates Mca-YVADAPK and Mca-APK-Dnp (AnaSpec, San Jose, CA, USA), respectively, according to our previous report [26]. The assay was performed in a microquartz cuvette using 20 μL of diluted kidney homogenates and 2 μL of the fluorogenic substrates (stock concentration: 4 mM ACE substrate/1.5 mM ACE2 substrate) in ACE or ACE2 assay buffer (a total of reaction volume is 300 μL). The reaction was measured for 15 sec every 45 sec for 1 hour using a fluorescence reader at 330 nm/390 nm. Each sample was detected in duplicate and normalized to a positive control in the same plate. All samples were fitted and plotted using Grafit v. 4.0 (Sigma-Aldrich, St. Louis, MO, USA). Samples were also incubated with the above-mentioned reaction mixture in the presence of 1 μM captopril (Sigma-Aldrich; a specific ACE inhibitor) or 1 μM DX600 (AnaSpec; a specific ACE2 inhibitor) for the ACE and ACE2 activity assays, respectively.

### Measurements of chymase activity

Chymase activity was detected using the gold nanoparticles (AuNPs)-peptide probe developed by our laboratory (FITC-Acp-DRVYIHPFHLDDDDDC-AuNPs) [27,28]. A total volume 250 μL, including AuNPs-peptide probe (125 μL), reactive buffer (pH 8, TTC buffer) and kidney tissue homogenous extract, was incubated in a micro-quartz cuvette at 37°C for 15 min. The fluorescence intensity was recorded and analyzed at 515 nm at an excitation wavelength of 495 nm using a fluorescence spectrophotometer. The specific chymase inhibitor chymostatin (Sigma-Aldrich) was used in parallel samples to determine the accurate activity of chymase in kidney tissue.

### Renal Ang II and inflamatory cytokine measurements

The concentrations of renal Ang II and inflamatory cytokines in mouse kidneys were determined using mouse Ang II (Biocodon; Broadmoore, KS, USA), IL-6, TNF-α and TGF-β1 ELISA kits (Abcam, Cambridge, MA, USA). The levels in each samples were determined in the same volumes in duplicate. Measurements were performed according to the manufacturer's instructions. Briefly, tissue homogenates were incubated in 96-well ELISA plates with primary antibodies. Biotinylated antibodies were added, and the plates were washed and reacted with HRP-conjugated streptavidin. Tetramethylbenzidine (TMB) one-step substrate tablets were used for the detection of targets (Ang II, IL-6, TNF-α, and TGF-β1), and the results were measured at 450 nm using a micro-plate reader (Thermo Scientific, Waltham, MA, USA). The intensity of the color developed was inversely proportional to the target concentration in the samples.

### Histological determination

Kidney tissues were collected from the experimental mice, soaked in 10% formalin overnight, embedded in paraffin, and cut into 6-μm-thick sections on acid-pretreated slides for hematoxylin/eosin (H&E) staining to investigate leukocyte infiltration, which indicated inflammation, and periodic acid–Schiff (PAS) stain to examine lesions of tubular atrophy. The stained pathological sections were photographed using a digital camera mounted on a microscope. A computerized microscope equipped with a high-resolution video camera (BX 51; Olympus, Tokyo, Japan) was used for morphometric analysis.

### Biochemical analysis

Animal body weights were recorded daily or weekly during the experiments. Urine samples were collected weekly using metabolic cages (Tecniplast, Buguggiate, Italy) for 24 hr and stored at -30°C after centrifugation at 3,000 rpm for 15 min. Urine and plasma levels of creatinine, albumin and BUN were measured using a clinical chemical analyzer (#DRI-CHEM 3500; Fujifilm Medical, Tokyo, Japan) to evaluate mouse renal functions.

### Statistical analysis

All values are expressed as the means ± standard deviation (SD). One-way analysis of variance (ANOVA) tests were performed to evaluate differences between multiple groups. Significant data were further examined using post hoc tests to determine the significance between groups. A value of p < 0.05 was considered statistically significant.

## Results

### Physiological changes of mice with AAI-induced kidney injury

**Fig 1:**
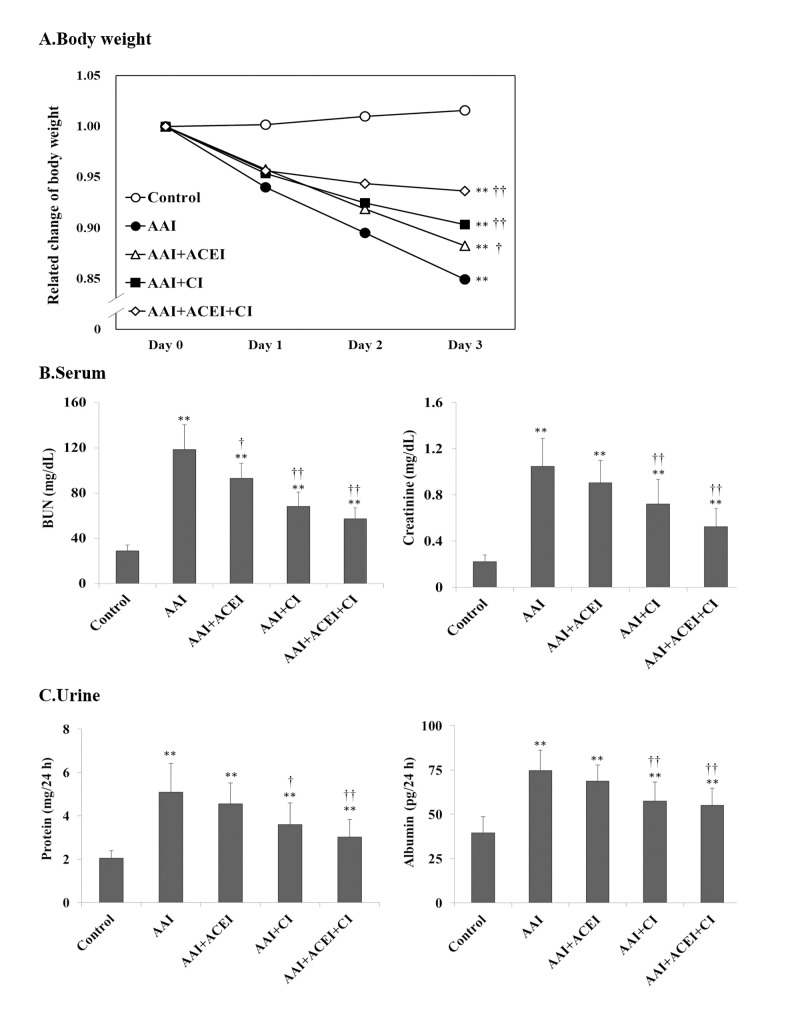
Body weights, serum and renal biochemical determinations in mice with AAI-induced acute nephropathy. Animal model of nephropathy was performed in C57BL/6 mice. The mice were treated with AAI via intraperitoneal (i.p.) injection, and the accumulated AAI dosage was 45 mg/kg BW (15 mg/kg BW per day for 3 days). Five groups of 9 mice per group were used. Mouse body weights were recorded daily (**A**), changes in serum BUN and creatinine (**B**) levels, and urine protein and urine albumin **(C)** levels were determined. All values are expressed as the means ± SD of each group (n = 9). ** indicates *p* < 0.01 compared to the Control group; † and †† indicate *p* < 0.05 and *p* < 0.01 compared to the AAI group, respectively.

Body weights of C57BL/6 mice were recorded daily during AAI treatment. The body weights of Control group mice treated with PBS exhibited no significant changes. However, body weights decreased significantly after 3 days of AAI treatment, and significantly recovered after ACEI, CI and ACEI+CI treatment compared to the AAI treatment group (**Fig 1A**). The decrease in body weight was the result of a significant decrease in food and water intake after AAI treatment. This physiological change reflects the effect of AAI treatment on organ damage.

Biochemical analysis revealed significantly increased serum BUN and creatinine concentrations in mice treated with AAI for 3 days during the progression of acute kidney injury. The levels of serum BUN and creatinine were lower in the groups treated with AAI and ACEI, CI and ACEI+CI treatment than the AAI group (**Fig 1B**). Urine protein and albumin levels were also significantly elevated in mice after AAI treatment. The concentrations of urine protein and albumin decreased significantly after CI and ACEI+CI treatment compared to the AAI group (**Fig 1C**). Increased serum BUN and creatinine and proteinuria are related to kidney dysfunction, and our results indicated that AAI caused severe renal impairment in the experimental animals.

### Pathological findings in kidney

**Fig 2:**
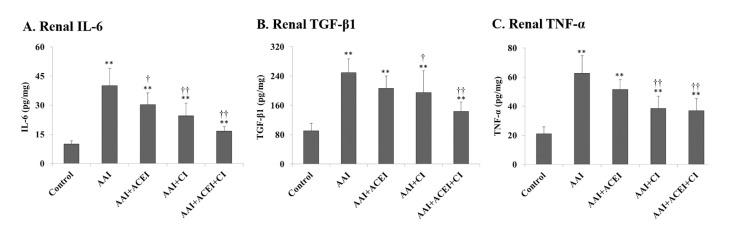
Renal inflammatory cytokine determinations in mice with AAI-induced acute nephropathy. Changes in renal IL-6 (**A**), TGF-β1 (**B**) and TNF-α (**C**) levels were determined. All values are expressed as the means ± SD from each group (n = 9). ** indicates *p* < 0.01 compared to the Control group; † and †† indicate *p* < 0.05 and *p* < 0.01 compared to the AAI group, respectively.

Analysis of renal immunocytokine expression confirmed that AAI treatment induced renal inflammation in mice. The levels of renal IL-6, TGF-β1 and TNF-α in mice treated with AAI were approximately 4.1- (*p* < 0.01), 2.7- (*p* < 0.01) and 3.0-fold (*p* < 0.01) higher than the control mice, respectively **(Fig 2)**. The inhibition of Ang II generation also efficiently reduced the expression and secretion of renal immunocytokines. The effect of chymase inhibition was larger than ACE inhibition.

**Fig 3:**
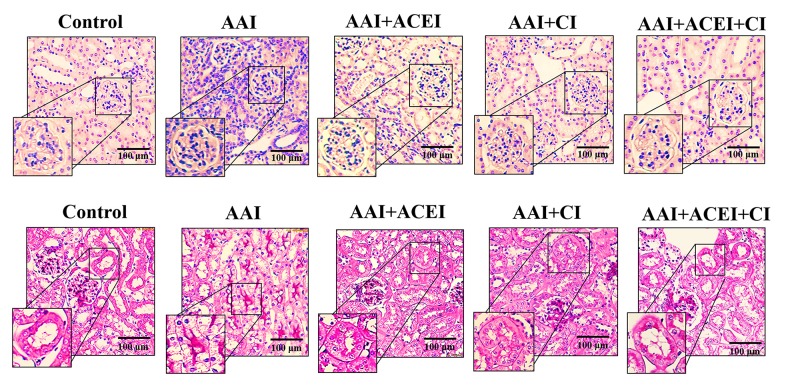
Pathological features of AAI-induced kidney injury revealed by light microscopy. The upper panels show H&E staining and leukocyte infiltration indicative of the inflammation induced by AAI treatment and the alleviation of this response by ACEI, CI and the combination of ACEI and CI (ACEI + CI) treatments. The lower panels show PAS staining and the lesions of tubular atrophy induced by AAI treatment, and the alleviation of lesions by ACEI, CI and ACEI + CI treatments.

Pathological staining of kidney tissues revealed significant interstitial infiltration of inflammatory cells and severe tubular atrophy in the AAI group compared to the Control group. These results indicate the AAI induced acute nephropathy in mice. Kidney tissues from the AAI-treated mice that received ACEI, CI and ACEI+CI treatments exhibited significantly reduced inflammatory cells and lesions of tubular atrophy. These results indicate a dramatic amelioration of acute kidney injury in mice treated with ACEI and/or CI treatments compared to AAI-treated mice (**Fig 3**).

### Renal RAS-related enzyme activities and Ang II levels

**Fig 4:**
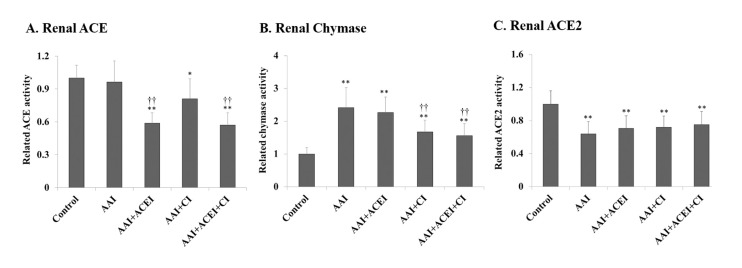
Changes in RAS enzyme activity in kidneys of mice with AAI-induced acute nephropathy. Renal ACE (**A**), chymase (**B**) and ACE2 (**C**) activities were determined, and the related activity levels are shown as levels relative to the Control group. All values are expressed as the means ± SD from each group (n = 9). * and ** indicate *p* < 0.05 and *p* < 0.01 compared to the Control group, respectively; †† indicates *p* < 0.01 compared to the AAI group.

**Fig 5:**
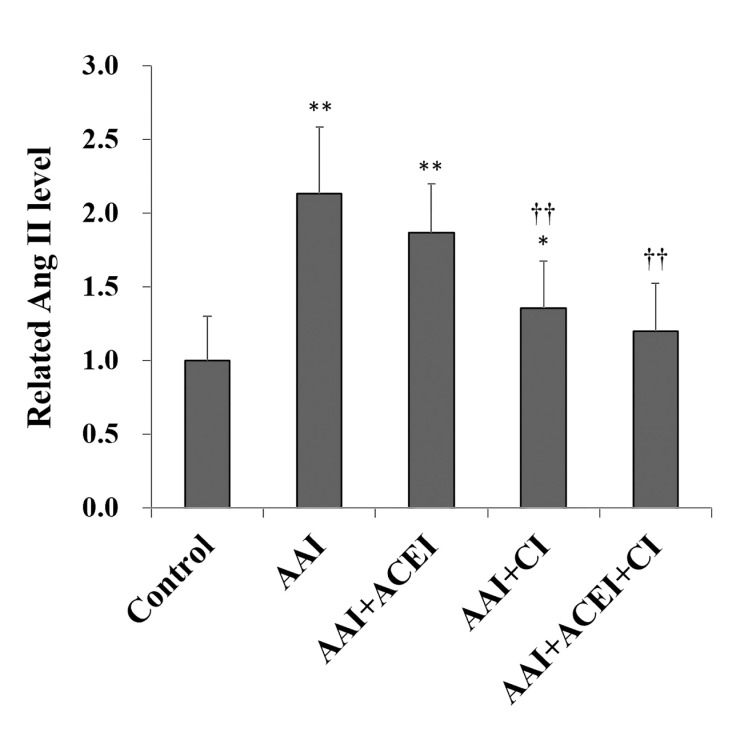
Changes in Angiotensin II (Ang II) in kidneys of mice with AAI-induced acute nephropathy. Renal Ang II levels were determined, and the related activity levels are shown as the levels relative to the Control group. All values are expressed as the means ± SD from each group (n = 9). * and ** indicate *p* < 0.05 and *p* < 0.01 compared to the Control group, respectively; †† indicates *p* < 0.01 compared to the AAI group.

Renal chymase level was significantly increased 2.4-fold after AAI treatment (*p* < 0.01). ACE2 level was significantly decreased 0.6-fold (*p* < 0.01), and renal ACE activity was slightly decreased compared to the Control group (**Fig 4**). The levels of renal ACE were significantly decreased in ACEI and ACEI+CI treatment groups (**Fig 4A**), and chymase activities were significantly reduced in AAI-treated mice treated with CI and ACEI+CI treatments (**Fig 4B**). Renal ACE2 levels were not significantly altered after ACEI, CI and ACEI+CI treatments (**Fig 4C**). These results demonstrated that the inhibition of Ang II production did not affect renal ACE2 expression. Renal Ang II levels reflect changes in the profile of these RAS enzymes and were significantly increased in AAI-treated mice (i.e., AAI group), the AAI/ACEI and AAI/CI groups compared to the Control group. Ang II levels decreased significantly after CI and ACEI+CI treatment in AAI-treated mice (*p* < 0.01). Notably, no significant difference in Ang II levels was observed between the AAI/ACEI+CI and Control groups (**Fig 5**).

### Molecular expression in the involved signaling pathways

**Fig 6:**
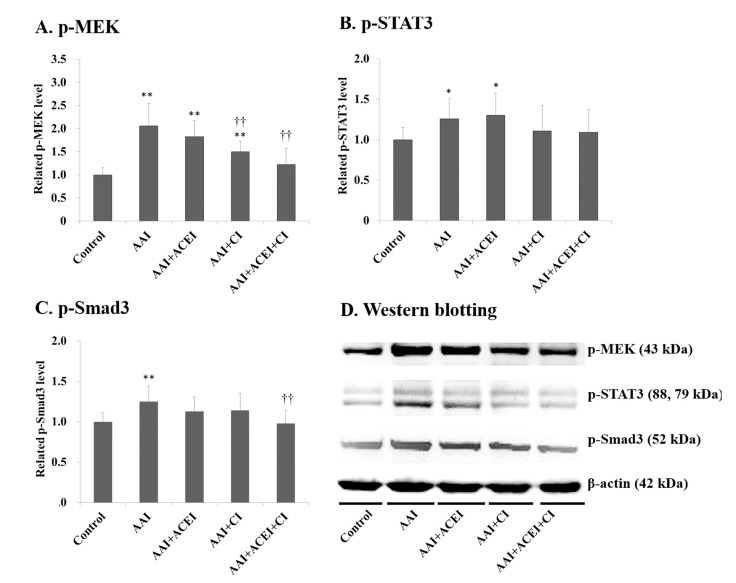
The level of inflammatory signaling pathways in the kidneys of mice with AAI-induced acute nephropathy. The expression of p-MEK (**A**), p-STAT3 (**B**) and p-Smad3 (**C**) in kidney tissues was determined using Western blotting and representative results of Western blotting are shown (**D**). All values are expressed as the means ± SD from each group (n = 9). * and ** indicate *p* < 0.05 and *p* < 0.01 compared to the Control group, respectively; †† indicates *p* < 0.01 compared to the AAI group.

**Fig 7:**
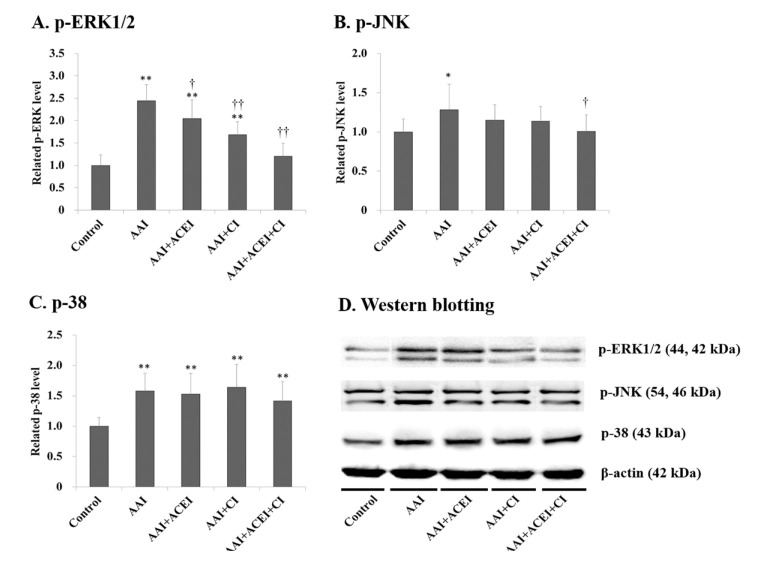
The MAPK signaling pathways in the kidneys of mice with AAI-induced acute nephropathy. The expression of p-REK1/2 (**A**), p-JNK (**B**) and p-38 (**C**) in kidney tissue was determined using Western blotting, and representative results of Western blotting are shown (**D**). All values are expressed as the means ± SD from each group (n = 9). * and ** indicate *p* < 0.05 and *p* < 0.01 compared to the Control group, respectively; † and †† indicates *p* < 0.05 and *p* < 0.01 compared to the AAI group, respectively.

The levels of renal p-MEK, p-STAT3 and p-Smad3 expression increased significantly after AAI treatment by 2.06- (*p* < 0.01), 1.26- (*p* < 0.05) and 1.25-fold (*p* < 0.01), respectively (**Fig 6**). Notably, CI and ACEI+CI treatment significantly decreased AAI-induced p-MEK expression (**Fig 6A**). However, the inhibition of Ang II generation did not reduce AAI-induced p-STAT3 and p-Smad3 expression (**Fig 6B** and **6C**). The expression levels of p-ERK, p-JNK and p-38 in the MAPK signaling pathway were also significantly increased in AAI-treated mice by 2.44- (*p* < 0.01), 1.28- (*p* < 0.05) and 1.58-fold (*p* < 0.01), respectively (**Fig 7**). The levels of AAI-induced p-ERK expression were significantly reduced after ACEI, CI and ACEI+CI treatments of AAI-treated mice (**Fig 7A**). However, Ang II blocker treatment only slightly reduced AAI-induced p-JNK and p-38 levels (**Fig 7B** and **7C**).

## Discussion

The present study induced acute kidney injury in mice using a high dosage of i.p. AAI, and the biochemical, pathological and immunological findings demonstrated that AAI-related acute nephropathy was induced. The AAI-induced acute nephropathy was associated with inflammation, RAS dysregulation and abnormally increased renal Ang II. This study is the first demonstration that modulation of Ang II signaling, especially chymase inhibition, alleviated AAI-induced kidney injury.

AAI is nephrotoxic and causes upper urinary tract urothelial carcinoma and kidney fibrosis [29]. AAI-induced acute kidney injury in a mouse model was recently identified by impaired expression of profibrotic genes and proteins associated with their downstream target genes in renal tissues [30,31]. Generally, dosage of 1 to 5 mg/kg BW of AAI administration is used to induce chronic kidney disease [9]. A dosage of AAI > 10 mg/kg BW [32] and a single AAI dose of 10, 20 and 30 mg/kg BW [33] were used in mouse models of acute kidney injury. LD_50_ values in mice after oral and intravenous (i.v.) administration of AA are 60–106 mg/kg BW (survival time of 1–15 days) and 38–70 mg/kg BW (survival time of 1–13 days), and female mice are more tolerable to acute AA toxicity than male mice [34]. The effects of AAI-induced kidney toxicity in experimental mice are variable and depend on mouse species, age and gender. Our study used female C57BL/6 mice, and AAI was injected i.p. Therefore, an LD_50_ > 70 mg/kg BW was expected. Our preliminary results revealed that mice did not die until the 6th day after 45 mg/kg BW AAI treatment. Therefore, an accumulated AAI dosage of 45 mg/kg BW (15 mg/kg BW per day for 3 days) was administered to induce acute kidney injury in mice.

Ang II in RAS is a key factor in the inflammatory and fibrotic responses in kidney diseases [35,36]. Abnormally increased Ang II may be involved in the renal fibrotic process because of its behavior as a proinflammatory cytokine. Adequate Ang II regulation may prevent kidney inflammatory injury and fibrosis progression [37]. Chymase is the main ACE-independent pathway of Ang II generation in mice, and it is involved in blood pressure regulation during ACE inhibitor therapy [38]. Chymase activity is associated with glomerulosclerosis and renal interstitial fibrosis in animal models [39]. Animal studies suggest that chymase inhibition is beneficial for the treatment of hypertensive, diabetic and inflammatory nephropathies [39]. Chymase is primarily released during tissue injury or inflammation, and it promotes tissue remodeling [40]. Renal ACE activity in the AAI group was not significantly altered, and renal chymase activity was markedly increased in our study. The levels of renal Ang II indicated changes in the profile of these RAS enzymes, and a noticeable increase in renal Ang II was observed in AAI-induced acute nephropathy. The combination of ACEI and CI treatment significantly reduced Ang II levels and alleviated acute kidney injury in AAI-treated mice. These results suggest that the chymase-dependent Ang II axis is a critical pathway in AAI-induced acute nephropathy.

Our results demonstrated significantly higher levels of renal p-MEK, p-STAT3 and p-Smad3 in AAI-treated mice. However, only the inhibition of Ang II generation with ACEI, CI, and ACEI+CI significantly mitigated AAI-induced p-MEK activation. MEK is the most representative molecule in the MAPK signaling pathway, and it is activated by the oxidative and inflammatory stresses induced by excess Ang II [41]. Inflammation triggered by IL-6 and TGF-β induces p-STAT3 and p-Smad3, respectively [42,43]. TGF-β leads to kidney tissue fibrosis via inhibition of matrix degradation and stimulation of myofibroblast activation. Smad3 is a key factor in this process because deletion of Smad3 protects against AAN [3]. Our results explain why the inhibition of Ang II production significantly reduced active p-MEK but minimally reduced AAI-induced p-STAT3 and p-Smad3 levels. These results confirm the multiple mechanisms of tissue damage induced by AAI treatment [30,31,36]. Multiple mechanisms of AA-induced cytotoxicity are well defined, such as increased oxygen stress, inflammation, fibrosis, and the formation of DNA adducts [3]. The damage mechanism also includes an imbalance in RAS, which was demonstrated in our study. Therefore, these results explain why treatments with ACEI, CI and ACEI/CI ameliorated, but did not completely prevent, the toxic effects of AAI.

ERK, JNK and p38 are three major members of the MAPK signaling pathway [44]. However, Ang II blockade with ACEI, CI, and especially ACEI+CI only significantly mitigated active p-ERK in AAI-treated mice. This result suggests that AAI-induced acute nephropathy involved the p-ERK signaling pathway, which was associated with abnormally excessive Ang II formation. Numerous stimuli activate the ERK signaling pathway, which is correlated with the regulation of survival, differentiation, proliferation, mitosis, and apoptosis [45]. p-ERK plays a crucial role in the oxidation-dependent axis that results in kidney impairment [46], and it is activated in proliferative glomerulonephritis [47] and unilateral ureteral obstruction-induced renal fibrosis in rats [31,45]. p-MEK is an upstream regulator in the ERK signaling pathway [48]. Therefore, we demonstrated that AAI-induced acute kidney injury was associated with an imbalance of the RAS axis, which generated excess Ang II and p-MEK/p-ERK signaling activation. We observed a dramatic elevation of the p-MEK/p-ERK1/2 signaling pathway in the AAI group, which may be related to renal Ang II levels, and the combination treatment of ACEI and CI reflected the reduction of the AAI-activated signaling pathway and the changed profile of renal Ang II levels. This hypothesis is consistent with the previous studies. Qin et al. [49] reported that Ang II deteriorated renal interstitial fibrosis via the induction of oxidative stress and the ERK/MAPK signaling pathway. Liu et al. [50] indicated that Ang-II exacerbated glomerulosclerosis via enhancing p-ERK in chronic kidney disease.

## Conclusion

The experimental mice treated with a high dose of AAI exhibited renal inflammation and acute nephropathy. RAS dysregulation was associated with AAI-induced nephropathic progression and abnormally increased renal Ang II. Our results suggest that chymase plays a role in pathogenesis, and the AAI-induced chymase-Ang II axis exacerbated kidney injuries via the p-MEK/p-ERK1/2 signaling pathway. Therefore, the lowering of Ang II levels using inhibitors and specific chymase inhibition of the Ang II-generating pathways may effectively mitigate AAI-induced acute kidney injury. The present study elucidates the role of RAS in the pathogenesis of AAI-induced acute nephropathy. However, further research is needed to confirm whether the ACEI and CI combination treatment is a more potent renoprotective therapy for AAI-induced acute nephropathy.
